# Neural architecture search applying optimal stopping theory

**DOI:** 10.3389/frai.2025.1643088

**Published:** 2025-09-23

**Authors:** Matthew Sheehan, Oleg Yakimenko

**Affiliations:** Department of Systems Engineering, Naval Postgraduate School, Monterey, CA, United States

**Keywords:** neural architecture search, Markov decision processes, automated machine learning, optimal stopping theory, secretary problem, Markov time

## Abstract

Neural architecture search (NAS) exploration requires tremendous amounts of computational power to properly explore. This makes exploration of modern NAS search spaces impractical for researchers due to the infrastructure investments required and the time needed to effectively design, train, validate, and evaluate each architecture within the search space. Based on the fact that early-stopping random search algorithms are competitive against leading NAS methods, this paper explores how much of the search space should be explored by applying various forms of the famous decision-making riddle within optimal stopping theory: the Secretary Problem (SP). A total of 672 unique architectures, each trained and evaluated against the MNIST and CIFAR-10 datasets over 20,000 runs, producing 6,720 trained models confirm theoretically and empirically the need to randomly explore ~37% of the NAS search space until halting can occur for an acceptable discovered neural architecture. Additional extensions of the SP investigated include implementing a “good enough” and a “call back” feature; both further reduce exploration of the NAS search space to ~15 and 4%, respectively. Each of these investigations were further confirmed statistically upon NAS search space populations consisting of 100–3,500 neural architectures increasing in steps of 50, with each population size analyzed over 20,000 runs. The paper details how researchers should implement each of these variants, with caveats, to balance computational resource costs and the desire to conduct sufficient NAS practices in a reasonable timeframe.

## 1 Introduction

Neural architecture search (NAS), the process of automating architecture engineering (Elsken et al., 2019), results in state-of-the-art model performance as tied to its architecture design. It is typically executed over a vast search space with billions of design options to choose from and compare (Hu et al., 2020). The pioneering work by Stanley and Miikkulainen (2002), Zoph and Le (2016), and Baker et al. (2017) proposed algorithms that could design novel machine learning (ML) architectures, increase ML algorithm learning rates, and even outperform state-of-the-art models of the time. These deep learning successes proliferated NAS research into image classification (Huang et al., 2019; Chen et al., 2019); multi-objective genetic algorithm optimization (Lu et al., 2018), adversarial ML (Gong et al., 2019), autonomous driving (Cheng and Bender, 2019), natural language processing (NLP) (Ding et al., 2020), and activity prediction (Pellatt and Roggen, 2021), to name a few. NAS has proven itself, time and time again, as a viable method when the search for optimal model performance is dependent upon the model’s architecture building blocks and their configuration (Ying et al., 2019).

Unfortunately, the goals of researchers to discover novel neural architectures resulting in improved model performance are fundamentally at odds with the goals of engineers to take said discovered models and productionize them for market deployment. For researchers, the desire to find ever greater model performance leads to an exponential growth of the learning parameter count and, consequently, the required processing power (Thompson et al., 2020). Modern NAS search spaces now easily exceed 10^20^ solutions (Smithson et al., 2016). In February 2020, Microsoft introduced the largest NLP model with 17 billion learning parameters. In May 2020, OpenAI surpassed this record by releasing an NLP model with 175 billion learning parameters. Not to be outdone, in January 2021, Google introduced the current NLP model record with 1.6 trillion learning parameters. On average, notable machine learning models created in 2023 through 2024 contain over 43 billion learning parameters and have training datasets surpassing 1.19 trillion objects (Epoch AI, 2025).

This data points to a concerning trend: effective exploration of modern NAS search spaces are increasingly inaccessible to most researchers due to prerequisite requirement of having access to expensive and powerful computational hardware if a search space is to be explored in a meaningful capacity with time as a constraint. For example, GPT-4, produced by OpenAI, costs an estimated $78 million to train, and Gemini Ultra, produced by Google, costs an estimated $191 million to train and required 50 billion peta-floating point operations per second (peta-FLOPS) of computational power (Maslej, 2024). In fact, a 2024 analysis shows the costs of developing and training frontier AI models have continued to rise at the rate of 2.4x per year since 2016 with costs expected to eclipse a billion dollars by 2027 Cottier et al. (2024). Adding insult to injury, even with robust hardware, novel neural architecture discovery may take months of computational time to complete (Zoph et al., 2018).

Due to the pressures researchers and engineers face to make use of current organizational infrastructure, limit their expenses toward new infrastructure, and compress their development timelines to delivery models for production and deployment, hidden NAS dark patterns have emerged. Practitioners increasingly rely on model designs rooted in familiar architectural paradigms, favoring limited test case development, established problem-solving precedents, and trending ML model traits—an approach that may constrain the exploration of novel neural architectures (Ren et al., 2021). Thus, practitioners need an effective way to balance the rapid discovery of novel neural network (NN) architectures with their limited access to high performance computing infrastructure.

To address this, researchers have been attacking the optimization and implementation challenges presented by NAS through its four main aspects: search space, model construction, model training/evaluation, and search strategy. Advances in simplifying the global search space into multiple modular search spaces have shown a significant reduction in the size of the search space (Zoph et al., 2018). Paired with substantial efforts to improved search space quality (Radosavovic et al., 2019) and design (Tan and Le, 2019; Guo et al., 2020), state-of-the-art model performance is achievable without using knowledge distillation or weight pruning techniques (Ci et al., 2021). In the areas of model construction and model training/evaluation, the practices of NN architecture recycling (Ren et al., 2021; Sun et al., 2023), and incomplete training (Wu and Tsai, 2024) embody the proverb “if it ain’t broke, do not fix it.” Both practices have helped to minimize the computational resources required by speeding up the processes to execute these functions. Using existing high-performing models as the starting point for further evolution and reducing complete model training through the implementation of shared model structures have also increased model formation speed, training, and performance prediction times (Ren et al., 2021).

The search strategy, claimed to be the “most widely-studied” aspect of NAS, is the critical mechanism used to discover a high-performing NN architecture within the search space and are typically categorized as black-box optimization techniques or one-shot techniques (White et al., 2023). Within the black-box optimization category, the heavy-weights are reinforcement learning, evolutionary/genetic algorithms, and Bayesian optimization; whereas within the one-shot category, the chief methods are hypernetwork and supernetwork techniques. Each of these search strategies have shown, at the time of their publication, to achieve state-of-the-art performance (Elsken et al., 2019; Ren et al., 2021; White et al., 2023; Chitty-Venkata et al., 2023; Xie et al., 2023; Xiao et al., 2020; Liu et al., 2022a; Liu et al., 2022b; Chauhan et al., 2023). Additionally, multiple search space strategies have been created and extended to help minimize computational resource impacts (Chen et al., 2019; Xu et al., 2020), to include “hardware-aware” solutions addressing hardware latency and power constraints (Ci et al., 2021). The choice of which search strategy to implement is based on multiple factors such as computational infrastructure access which is a challenge for reinforcement learning and evolutionary/genetic algorithms (Chauhan et al., 2023), search algorithm flexibility which challenges Bayesian optimization techniques (Jaafra et al., 2018; Klein et al., 2016), and confidence the search space was effectively explored and the architecture found is indeed highly-performing compared to others within the search space which is a topic of current debate for one-shot techniques and the assumptions inherent to their approaches (Yu et al., 2020a; Ci et al., 2021; Pham et al., 2018; Yu et al., 2020b; Pourchot et al., 2020; Zela et al., 2020; Zhang et al., 2020).

However, despite all these improvements, random search methods not only perform unexpectedly well in executing NAS (Yu et al., 2020a; Li and Talwalkar, 2020; Chen et al., 2018; Yang et al., 2020) but continue to be consistent with the performance of state-of-the-art NAS algorithms (Yang et al., 2020; Yu et al., 2019; Lindauer and Hutter, 2020) as well as a competitive baseline for hyperparameter optimization and early stopping algorithms against leading NAS methods (Li and Talwalkar, 2020). Research also shows random search performance can be greatly increase by paring it with “highly engineered” search space development practices (White et al., 2023), NN architecture design and training methods (Li and Talwalkar, 2020), performance estimation prediction processes (Abdelfattah et al., 2021; Yan et al., 2021), heuristically driven search techniques (Ottelander et al., 2021; White et al., 2021; Siems et al., 2020), and NN evolution strategies (Elsken et al., 2017).

Based on this stark reality, this paper presents a promising path forward by applying optimal stopping theory (OST) to NAS. Borrowing the solution to the notorious Secretary Problem (SP) and further extending it twice, this paper demonstrates these modified SP approaches to be an elegant solution to the aforementioned issues that NAS practitioners face. The paper empirically, with caveats, shows when the exploration and evaluation of a NAS effort should be halted revealing a “satisfactory” architecture to be used. Expensive subject matter expertise, computational resource usage, and ML model time-to-market deployment can all be significantly reduced by applying OST when engaging in NAS techniques.

This paper is not focused on improving NAS search strategies where the goal is to find the best performing NN architecture for a given: dataset, set of NN architecture parameters, class of NNs, or to conduct an “apples-to-apples” comparison to other non-random NAS search strategies (e.g., reinforcement learning-NAS, one-shot, zero-cost, evolutionary NAS, once-for-all search, and the like) (Chen et al., 2021; Wu et al., 2021; Chen et al., 2023; Guo et al., 2021). Instead, this paper focuses on the application of OST to NAS and shows how the solution to the SP, and its extensions, form a set guiding heuristics on when to halt a NAS effort that is agnostic to the design and size of search space and NN architectures within.

The main contributions of this paper can be summarized as follows:

1. Empirical proof that the solution to the SP is a viable NAS search strategy technique only requiring ~37% of the NAS search space to be randomly explored until halting may occur for an acceptable discovered NN architecture.
2. Extension of the SP solution through the implementation of a “good enough” (GEP) and “call back” (CBP) NAS evaluation feature improving the performance of the SP solution; thereby reducing the required coverage of the randomly explored NAS search space to ~15% and 4%, respectively.
3. Creation and validation of five equations to aid researchers in estimating computational resources requirements, scheduling timetables, bounding risks associated with poor NAS outcomes, and communicating cost-schedule-scope tradeoffs to senior management.
4. Release of datasets and source code (in a variety of programming languages) to execute the SP and its variants for NAS search space populations of 100 through 3,500, stepped at increments of 50.

Due to the nature of the SP, the findings of this paper may not hold if the rules of the SP are not adhered to or if paired with another non-random search strategy. However, the findings of this paper do confirm there is a high degree of confidence in discovering a high-performing NN model relative to the performance of other NN models within the search space. Thus, practitioners should apply this paper’s findings and guiding heuristics with “smart” search space design practices aligned to the problem to be solved.

The reminder of the paper is organized as follows. Section 2 introduces the OST through the SP and its specific applicability to NAS. Section 3 details the experimental setup and materials, followed by a detailed summary of the investigation into and analysis of applying OST via the SP, and its variants, to NAS, confirmed over multiple experiment iterations in section 4. Section 5 presents a discussion of the findings, potential benefits, caveats, considerations, and future directions to study. Finally, section 6 closes with a conclusion to encapsulate the useful discovery.

## 2 Optimal stopping theory and the secretary problem

In the discipline of mathematics, optimal stopping is the process of determining when it is best to terminate a task to maximize the desired results and expected rewards (Tsitsiklis and Roy, 1999). Knowing when to stop executing a task is immensely powerful as it allows an investigator to optimize the expenditure of limited resources and apply these resources to other high priorities. Due to this, OST has been applied to problems in a wide array of disciplines and sectors including financial derivative markets, lottery ticket purchasing strategies, gambling schemes, World War II military-industrial complex production plans, stock option valuations, dynamic programming solutions, human resource hiring methods, and even personal relationship match-making endeavors (Hill, 2009).

OST problems typically come in one of three flavors: decision theory, statistical sequential inference, and the statistical design of experiments. However, the control of random sequences and statistical decisions is invariably the objective to achieve to inform an interested party to cease the task at hand or halt sampling in a statistical inference problem (Weber, 1975). In stochastic processes, this ceasing or halting is known as the stopping time or the Markov time (*τ*), as an optimal stopping problem is a finite horizon Markov decision process (MDP). One famous decision-making riddle within finite horizon MDP problems is known as the SP.

The SP goes by many names: the fussy suitor problem, sultan’s dowry problem (Swanson, 2016), best choice problem, beauty contest problem, marriage problem (Porosinski, 1987), and game of Googol problem (Gnedin, 1994). The simplest form of the problem consists of the following characteristics as portrayed by Ferguson’s historical investigation into the solving of the classic brain teaser (Ferguson, 1989):

1. There is one secretarial position available.
2. The number *n* of applicants is known.
3. The applicants are interviewed sequentially in random order, each order being equally likely.
4. It is assumed that you can rank all of the applicants from best to worst without ties. The decision to accept or reject an applicant must be based only on the relative ranks of those applicants interviewed so far.
5. An applicant once rejected cannot later be recalled.
6. You are very particular and will be satisfied with nothing but the very best. (That is, your payoff is 1 if you choose the best of the *n* applicants and 0 otherwise.)

A hiring manager should adopt an interview strategy maximizing the chance of success in finding the best applicant by appropriately interviewing an “optimal” number of candidates. The probability of picking a candidate he would rate the best after interviewing *r* candidates out of pool of n candidates, P(*r,n*), can be presented as the following sum (Billingham, 2008; Freiberger, 2017):


(1) $$P(r,n)=\frac{r}{n}\sum_{i=r+1}^{n}\frac{1}{i-1}$$

To maximize the chances for success, the “optimal” number of candidates to interview before selecting the next best one relative to the previous should satisfy two obvious relations


(2) $$\begin{array}{c} P(r-1,n)<P(r,n)\\ P(r+1,n)<P(r,n) \end{array}$$

Substituting Equation 1 into these two inequalities and doing some simplifications results in


(3) $$\sum_{i=r+2}^{n}\frac{1}{i-1}<1<\sum_{i=r+1}^{n}\frac{1}{i-1}$$

where the right-hand side part of inequality corresponds to satisfying the first inequality of Equation 2, and the left-hand side part – to the second inequality of Equation 2.

**Figure 1:**
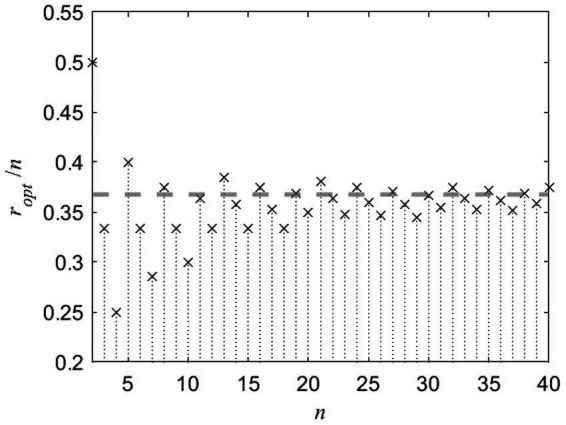
Optimal number of applicants to interview *r*_opt_ (normalized by the applicant pool size *n*) vs. *n*.

Both left-hand side sum and right-hand side sum in Equation 3, depend on *r*. For small *r*, both sums are greater than one so that the left-hand side part of inequality is violated. For large *r* – both sides are less than one resulting in violation of the right-hand side part of inequality. For given *n*, there is only one “optimal” *r* when both sides of inequality are satisfied. Figure 1 shows this best value as *r_opt_*/*n* against *n* (for the applicant pool size *n* of 3 through 40) featuring a quick convergence to some value.

**Figure 2:**
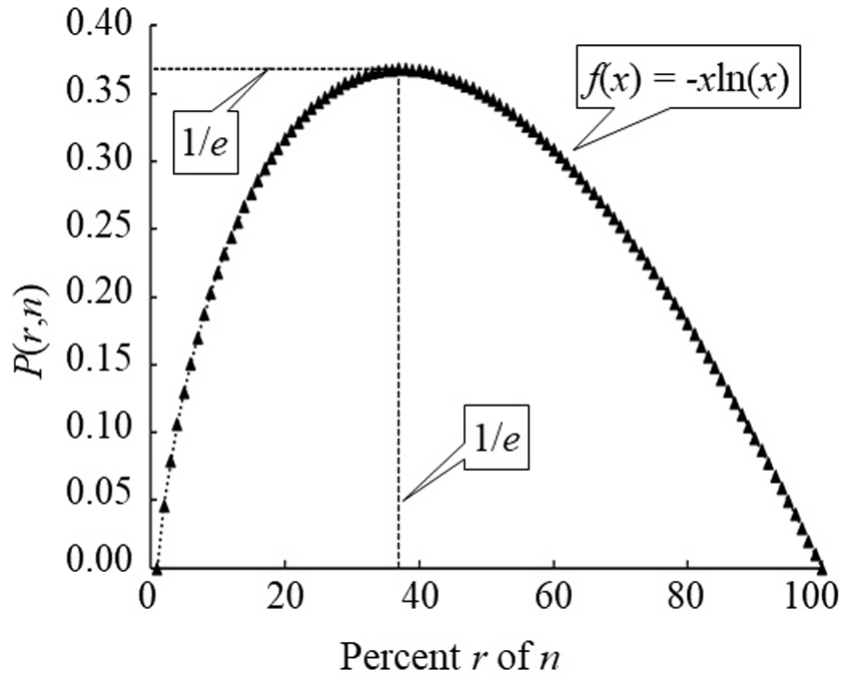
Probability of hiring the best applicant vs. percentage of applicants interviewed and rejected (*r*) within applicant pool size (*n*) (for large *n*).

This value can be derived analytically and corresponds to $r_{\mathrm{opt}}/n\approx e^{-1}\approx 0.368$. Hence, for the SP the best stopping or prime Markov time, referred to as the optimal policy (τ_*π*_) as defined by the problem’s stopping rule, converges to $\tau_{\pi }\approx 0.368$ without regard to the number of applicants *n* for large *n* (Figure 2). That is, the hiring manager should interview *r* and reject ~37% of the total applicants *n* and then select the next relatively best one for the position. The hiring manager starts to experience diminishing returns with each subsequent interview past this amount (Figure 2). This result has been developed, confirmed, extended, and generalized by many probabilists and statisticians, showing its versatile application to many probability optimization problems (Lindley, 1961; Dynkin, 1963; Chow et al., 1964; Gilbert and Mosteller, 1966).

With early-stopping random search algorithms being competitive against leading NAS methods, the application of a finite horizon MDP to govern the halting of a NAS endeavor is logical. While applying OST may not discover the highest-possible performing model within the NAS search space, it will significantly limit the need and time of computational resources. To confirm this theory, the original SP and two extensions were empirically investigated. This investigative process includes creating an NAS design space, training and evaluating each model independently to build a fully informed search space, applying the rules of the SP and its extensions to this search space, and finally analyzing the results. The design space was trained and evaluated against two datasets five times from which the SP and its extensions were played out 20,000 times against. To further confirm this paper’s findings, the size of the search space was modified with representative NN architectures totaling 100–3,500 in steps of 50 played over 20,000 times each.

For the remainder of the paper the following variants of the SP will be referred to as

- Classical secretary problem (CSP)—the original SP as described above with an optimal policy of 37% (*τ_π_* ≅ 0.37).
- Modified secretary problem (GEP)—an extension of the CSP where a model performance threshold and/or objective is used to deem a found model “good enough” allowing for early NAS halting when compared to the CSP’s optimal policy.
- Modern secretary problem (CBP)—an extension of the CSP and GEP where a model performance threshold and/or objective is used in conjunction with the capability to recall, or “call back,” any previously evaluated model.

## 3 Experiment materials and methods

To evaluate the effectiveness of the CSP and its variants as applied to NAS, a fully informed design and search space was built to ensure the experiment met computational and statistical power tractability requirements. The design space degrees of freedom for the NN building block components were limited to the values outlined in Table 1. The design space was further confined with restrictive combinations placed on the mixing of different input and hidden layer activation functions, optimizer algorithms/methods, and number of nodes per fully connected hidden layer. The total number of unique NN model architectures within the NAS search space was 672.

**Table 1:** Experimental design-space parameters.

ANN class	Parameter	Range	Steps	Sequence
Feedforward multilayer perceptron (MLP)	Number of fully-connected layers	1–4	4	(1, 2, 3, 4)
	Nodes per fully-connected layer	64–1,024	6	(64, 128, 256, 512, 768, 1,024)
	Activation function^1^	Rectified Linear Unit (ReLU), Exponential Linear Unit (ELU), Hyperbolic Tangent (TanH), or Logistic (Sigmoid)	4	N/A
	Optimizer algorithm/method	Root Mean Square Propagation (RMSprop), Adaptive Moment Estimation (Adam), Stochastic Gradient Descent (SGD), Adaptive Gradient Algorithm (AdaGrad), Adaptive Delta (AdaDelta)^2^, Adaptive Maximum (AdaMax)^3^, or Nesterov and Adam (Nadam)	7	N/A
	Batch size	Dataset Dependent^4^	1	N/A
	Training epochs	10,000^5^	1	N/A
	Output layer activation function	Softmax^6^	1	N/A
	Dropout frequency	80% Retain, 20% Dropout	1	N/A

1 The output layer uses the Softmax (described in table footnote 6) activation function for all networks within the search space.

2 The abbreviation of AdaDelta is not spelled out; however, its creator refers to a ∆x_t_ function which gives the AdaDelta method (Zeiler, 2012).

3 The abbreviation for AdaMax is not spelled out; however, its creators refer to a max() function which gives the AdaMax method (Kingma and Ba, 2015).

4 For both MNIST and CIFAR-10, the number of training and testing images is 60,000 and 10,000, respectively.

5 The training and scoring of each model uses an extensible program-code-template to halt training when five epochs have passed with no improvement (Keras Application Programming Interface Callback Object EarlyStopping Class with Patience argument set to five).

6 The abbreviation for Softmax is not spelled out; however, the “soft” part of the term describes a function which is continuous and differentiable. This function provides a “softer” version of the ArgMax function, which is the opposite of the ArgMin function. The ArgMin function minimizes the distance between an input point and its reconstruction using a measure which gives the size of a vector, known as the norm. It is also known as “softargmax” (Goodfellow et al., 2016).

To ensure a diverse, non-bias experiment was performed in evaluating the effectiveness of the SP variants, two different popular benchmark datasets were chosen: the Modified National Institute of Standards and Technology (MNIST) and the Canadian Institute for Advanced Research, 10 Classes (CIFAR-10). Both datasets are image collections; however, each dataset provides significant performance differences based on the NN building block components outlined in Table 1. This was done intentionally as the goal of the experiment was not to find the highest-possible performing NN architecture within the search space, but to baseline all the possible NN architectures within the search space and then test the effectiveness of applying the optimal policy of the CSP and its variants to the mechanics of NAS.

This experiment made use of the Department of Defense’s (DoD) High Performance Computing (HPC) Modernization Program and two separate local non-HPC systems. Of the four DoD HPCs, the individual unclassified HPC system utilized was known as Gaffney; a Hewlett Packard Enterprise Silicon Graphics, Incorporated 8,600 scalable, high-density cluster compute system featuring liquid cooling, 154 terabytes of memory, 5.5 petabytes of formatted parallel disk storage, and has a peak performance of 3,029 trillion floating-point operations per second (United States Department of the Navy, 2021). Source code for training, testing, and evaluating the NAS search space was developed and tested on a local non-HPC machine and then ported over to the Gaffney HPC system for training, testing, and storage at-scale.

Data analysis was performed on two separate local non-HPC systems. These two systems executed the vast majority of: algorithm validation, experiment source code development, experiment verification, experimental data management and compression, programming language translation, and data visualization. In total, 6,720 individual NN model performance data points and structures were each captured at the post training phase. These 6,720 NN models were trained and tested against 130,000 images resulting in 436,800,000 data pipeline flows and 489,989,427,200,000 parameter adjustments. This resulted in each of the 6,720 NN models receiving a performance score for image classification accuracy. The performance score, five per a unique NN architecture per a dataset, were averaged to create two sets of “master” performance scores. Thus, 672 average performance scores per unique NN architecture were created for each of the two datasets.

Table 2 highlights and compares relevant performance measures and statistical features of each “master” performance score against each dataset. The image classification performance scores for each NN architecture contained within the NAS search spaces, tied to each dataset, varied greatly. This met the goals of this paper to ensure the application of the SP, and its variants were independent of NN architecture and dataset selection.

**Table 2:** NAS search space networks performance and statistical features by dataset.

Dataset	Maximum performance	Minimum performance	Average performance	SD
MNIST	98.69%^1^	8.92%^2^	97.84%^3^	0.65%^4^
CIFAR-10	56.31%^5^	10.00%^6^	40.44%	12.40%

1 The best performing neural network architecture against this dataset consisted of 4 layers, 768 neurons per layer, using the ELU activation function, and the RMSPROP optimizer.

2 The worst performing neural network architecture against this dataset consisted of 3 layers, 1,024 neurons per layer, using the RELU activation function, and the ADAGRAD optimizer.

3 This statistical value was computed after discarding 12 outlier performance data points (1.79% of the total data collected). If this discarded data is included, the average performance becomes 96.27%.

4 This statistical value was computed after discarding 12 outlier performance data points (1.79% of the total data collected). If this discarded data is included, the standard deviation becomes 11.67%.

5 The best performing neural network architecture against this dataset consisted of 2 layers, 512 neurons per layer, using the ELU activation function, and the ADAMAX optimizer.

6 The worst performing neural network architecture against this dataset consisted of 4 layers, 1,024 neurons per layer, using the SIGMOID activation function, and the SGD optimizer.

With the two “master” performance score datasets built, each containing 672 NAS search space networks scores per experimental dataset, the two datasets were sorted from the highest performing architecture to the lowest performing architecture. Once sorted, each search space network entry was assigned a static key. This static key served as the identifier and performance rank *R_i_* [where *i* is the position of the NN within the performance rank list and *R*_1_ = *min* (*R*_1_,…, R*_n_*) for {*i*|*i* ∈ *n*∶ *i* ∈ (1, *n*)}] for each search space network as enumerated within each “master” performance score dataset. With a fully informed search space built, trained, and evaluated, these datasets were ready for the next step: investigating the application of OST to NAS.

## 4 Application and analysis of SP variants

To investigate the results of applying the CSP’s optimal policy to NAS using the created search spaces, the two “master” performance datasets static identification keys were shuffled, selected at random, and relatively ranked by their performance against previous selections until the CSP’s optimal policy (*τ_π_* ≅ 0.37) was achieved. This cycle was carried out 20,000 times to ensure the resulting analysis conducted had statistical significance and to build confidence in the data generated. The analysis of the modified (i.e., GEP or “good enough”) and modern (i.e., CBP or “call back”) SP variants also used the aforementioned shuffle, random selection, and relative rank process. The resultant analysis of each SP variant is detailed in the following subsections.

### 4.1 CSP application analysis

In testing the application of CSP’s optimal policy for NAS against the traditional rules of the SP over 20,000 cycles, it was found that the selection of the best performing NN model (*R*_1_), independent of dataset evaluated against, was selected 278% more often than the second best performing NN model (*R*_2_). With negligible selection difference between the datasets, the best performing NN model was selected: ~603% more than the third best performing NN model (*R*_3_), ~1,225% more than the fourth best performing NN model (*R*_4_), and ~2,335% more than the fifth best performing NN model (*R*_5_). The top 1% of performers (*R*_1-7_) contribute to almost 63% of the total population’s cumulative distribution function (CDF). After the top 1% of performers, each additional NN model’s contribution to the total CDF averages 0.05% (*R*_8-672_). More interestingly, the selection difference between NN models performing within the top 5% (*R*_1-34_) of the total population, regardless of dataset, produced almost identical selection results. When executing the rules of the CSP, each dataset (MNIST and CIFAR-10) produced almost identical results.

Through dynamic programming, real-time experimental measures were collected on the algorithm’s optimized decision making. This allowed the collection of four additional algorithm execution datasets: best of remaining (BOR), best of the rejected set (BORS), selected value (SV), and last in list (LIL). The BOR dataset refers to the highest-performing NN model left within the non-interviewed population after halting at a chosen Markov time, *τ*. The BORS dataset refers to the highest-performing NN model dismissed after being interviewed within the chosen *τ*. The SV dataset refers to the NN model rank selected at the end of executing a SP variant at the chosen *τ*. The LIL refers to the NN model rank which would be “interviewed” last as virtue of random selection.

With these measures, executing the CSP over 20,000 runs revealed the following: the best performing NN model (*R*_1_) was rejected 37.17% of the time; the LIL NN model was selected 37.23% of the time with an average rank consistent of normal distribution data behaviors (*R*_*n*/2_ = *R*_336_); 62.83% of time the best performing NN model (*R*_1_) still remained in BOR dataset; and 62.87% of time a lower performing NN model was selected over a better BOR model due to being found first and being comparatively better than the highest ranked model contained within BORS. On average, the BOR dataset rank was 1.58 with a standard deviation of 0.95. The BORS dataset rank had a mean of 2.69 with a standard deviation of 2.13. While both the BOR and BORS datasets had a minimum rank of *R*_1_, their maximum rank was *R*_11_ and *R*_21_, respectively. Meaning over 20,000 runs, if the NN model of *R*_1_ was not rejected, the average rank of the NN model selected was 2.03 with the lowest NN model being selected having a rank of *R*_18_. In fact, it was found that if the NN model of *R*_1_ was not rejected, the lowest NN model rank selected had an upper bound of (rank no worse than)


(4) $$R_{U}^{\mathrm{CSP}}\leq \sqrt{n}$$

**Figure 3:**
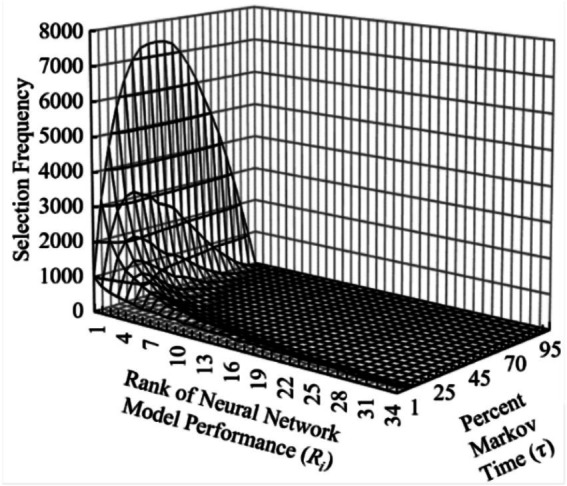
Three-dimensional surface plot demonstrating top NN model performance dominating selection frequency over increasing Markov time.

To further validate the CSP solution as applied to NAS, the percentage of NN models “interviewed” was tested over the total population spectrum in 1 % Markov time increments, *τ*_0.1–1.0_, where {*τ* ∈ ℝ∶ *τ* ∈ (0, 1)}. At each 1 % increment, the CSP was executed over 20,000 cycles with this new Markov time and analyzed. As expected, the top performing NN model (*R*_1_) was selected at the same rate predicted in the previously discussed solution to the CSP, i.e., ~37%. In comparing the selection frequencies for the top performing NN models over differing Markov times, it becomes apparent that the top 1 % (*R*_1-7_) performing NN models of the total population quickly dominate the selection frequency as the Markov time increases. To illustrate this, Figure 3 shows a surface plot of selection frequency vs. NN model performance rank vs. Markov time as a percentage of total population for the top 5 % performing NN models within the total population.

The goal of the CSP’s optimal policy is to maximize the success of selecting the highest ranked candidate (*R*_1_). NAS researchers applying the CSP’s rules and optimal policy will successfully discover the highest-performing (*R*_1_) NN model 37% of the time (*τ_π_* ≅ 0.37).

### 4.2 GEP application analysis

**Figure 4:**
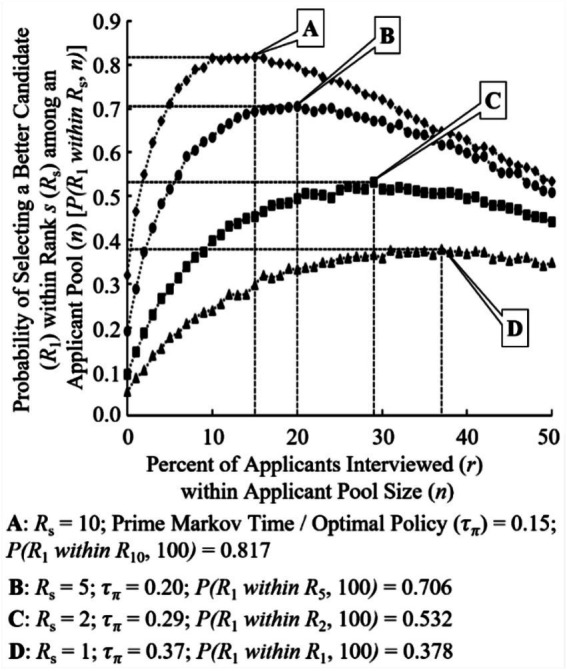
Probability of selecting a better candidate (*R_i_*) within rank *s* (*R_s_*) and associated optimal policy (*τ_π_*).

The first variant of the CSP brings into question what does a “good enough” candidate look like. This is especially important as the MNIST and CIFAR-10 datasets produced scores of “top-performing” NN models within single digit percentages of each other. While conducting NAS, a researcher may be able to halt the search early due to finding a “top performing” NN model that is “good enough” to satisfy the problem at hand. By taking this modified approach (GEP), the optimal policy of *τ_π_* ≅ 0.37 for the CSP can be further reduced. Figure 4 shows this for a “good enough” NN model within ranks *R_s_* (*s*) where *s* = 1, 2, 5, 10 and where {*s* ∈ ℤ∶ *s* ∈ [1, *s*]} and *R_s_* ⊆ *n*. As *s* within *R_s_* increases, more applicants within *n* are included resulting in a decrease in applicants to interview. That is, if a hiring manager or NAS researcher loosens their selection criteria to not only be interested in finding the best candidate/neural architecture, the percent of the required search space to interrogate falls off dramatically.

**Figure 5:**
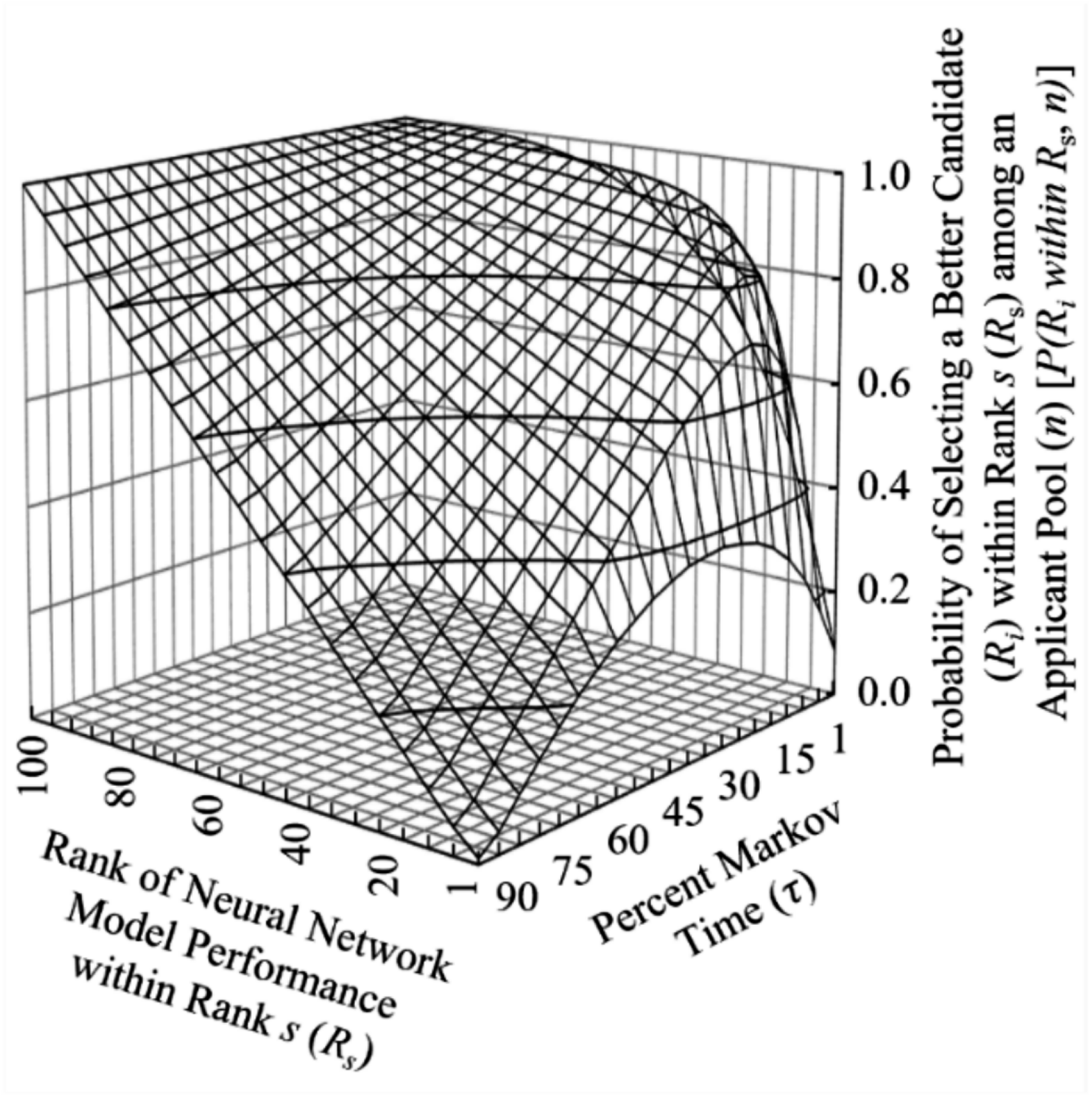
The three-dimensional surface plot demonstrating rapid increase of hiring a better candidate (*R_i_*) within rank *s* (*R_s_*) and resulting inverse relationship as s increases to Markov time decreasing.

Illustrating this dramatic increase in probability of successfully finding a candidate/neural architecture, Figure 5 shows the inverse relationship between the increase in *s* for *R_s_* and the decreasing Markov time needed. Similar to Figure 3, the top performing NN models of the total population (*n*) quickly dominate the probability of success when selecting an NN model within *R_s_* as *s* increases. By running this modified version of the SP with a total population of 100, two generalized equations can be realized. Approximation of the optimal policy (*τ_π_*) for a chosen NN model’s rank (*R_i_*) can be represented as


(5) $$\tau_{\pi }(R_{i})=\frac{1}{n}(\ln(n)\sqrt{n}{R_{i}}^{-\frac{\ln(n)}{\sqrt{n}}}-\frac{\sqrt{n}}{R_{i}}-\frac{\sqrt{n}\ln(n)-44}{R_{i}})+\frac{\rho }{100}$$

However, for larger values of *n* in Equation 5, the resulting approximation begins to deviate from experimental data collected. The inclusion of an error correcting value, ρ, where ρ≥2 as a conservative measure ensures the resulting optimal policy approximation calculated for a given NN model’s rank is greater than needed staving off inadequate search space exploration.

To estimate the probability of success in discovering a NN model of Rank *i* within a chosen Rank *s* $P(R_{i}\;\text{within}\;R_{s},n)$, the following relationship can be used:


(6) $$P(R_{i}\;\text{within}\;R_{s},n)=\frac{1}{e}(\frac{1}{2}\ln(R_{s})+1.04)$$

**Figure 6:**
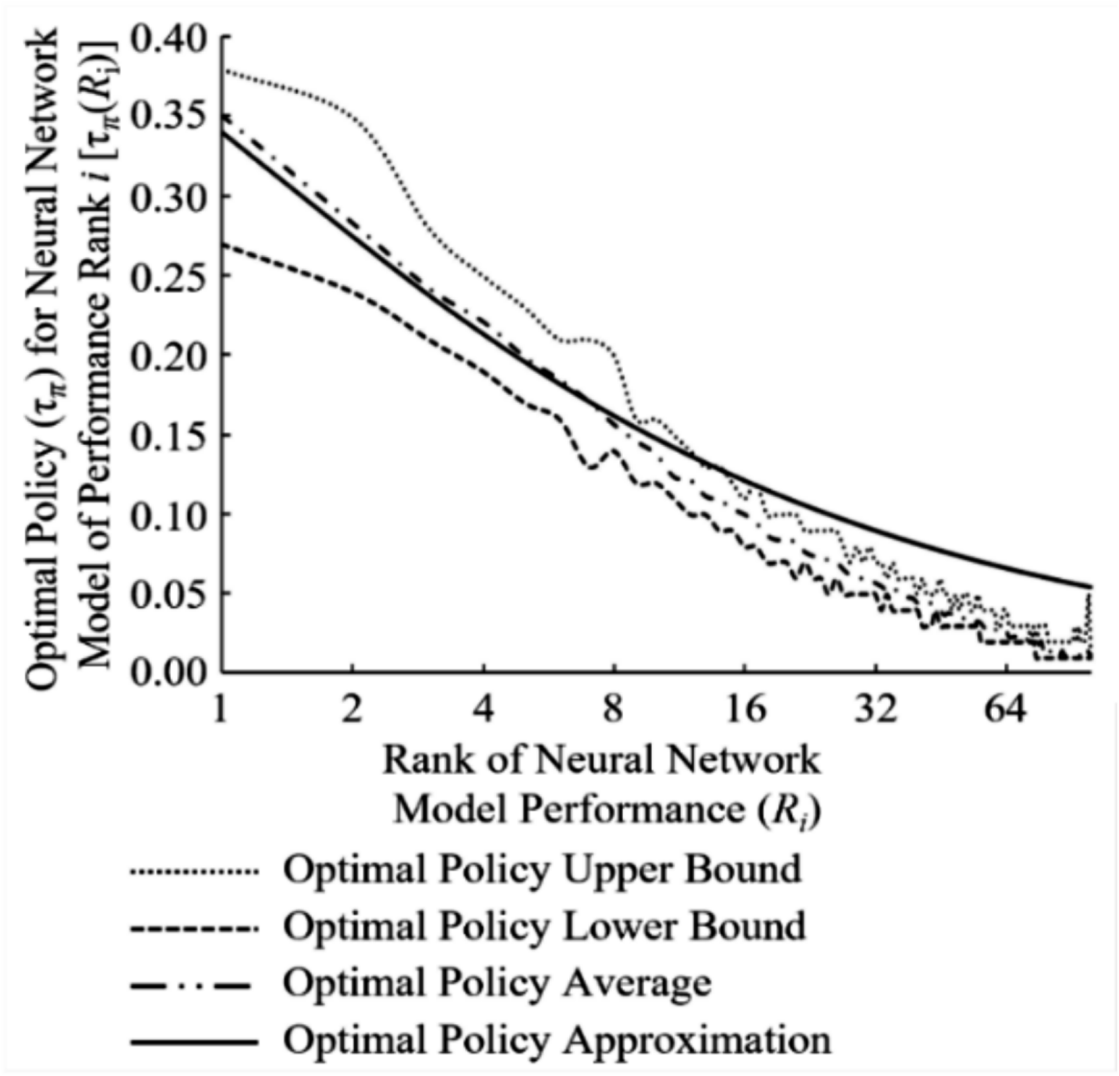
Experimental results verses equation approximation for optimal policy (*τ_π_*) of rank *i* (*R_i_*) as function of Markov time.

While Equations 5 and 6 were generalized to allow for use upon different total populations of *n*, there is some variability when using both as they are only rudimentary conjectures. The variability in Equation 5 is highlighted in Figure 6. Equation 5 approximates Figure 6’s optimal policy average line reasonably well (within +/− 3% absolute, +/− 1% on average) for all NN model ranks when the total population *n* is 100.

**Figure 7:**
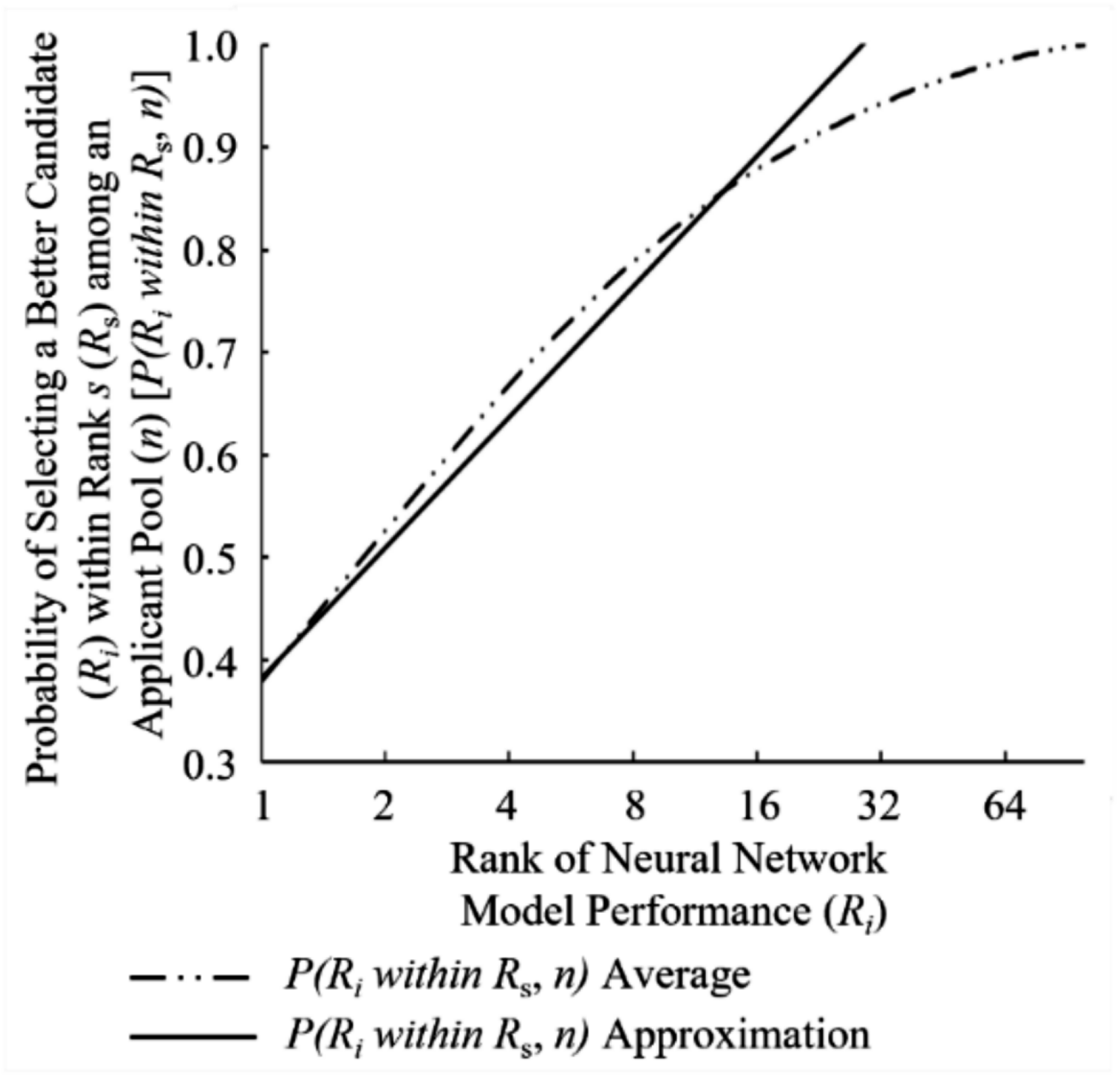
Experimental results verses equation approximation for probability of success selecting rank *i* (*R_i_*) within rank *s* (*R_s_*) where {*i*|*i* ∈ *n*: *i* ∈ [1, *s*]} and {*s* ∈ ℤ: *s* ∈ [1, *n*]}.

The variability in Equation 6 is depicted in Figure 7. Equation 6 approximates Figure 7’s probability of success in discovering a NN model of Rank *i* within Rank *s* $P(R_{i}\;\text{within}\;R_{s},n)$ average line well (within single digit percentage error) for NN model ranks less than *R*_39_ when the total population (*n*) is 100. However, for larger values of *n*, Figure 3 the resulting approximation begins to deviate past ranks above *R*_n*0.04_ to the tune of double-digit percentage error. Thus, the use of Equation 6 to approximate the probability of success in discovering a NN model of Rank *i* within Rank *s* average should be limited to cases where *s* ≤ 16 (*R*_16_).

If a researcher is to implement the “good enough” variant of SP (GEP), it is recommended that they at least explore and reject 15% of the NAS search space ($\tau_{\pi }\geq 0.15$). This provides the researcher a ~ 80% chance of success in discovering a NN model within $R_{10}$ or better. Table 3 shows this along with all successive ranks and their respective probabilities of discovery success inclusive of each other for two differing populations (*n* = 100 and 672). The variability of the results shown in Table 3 can be accounted for in the requirement to round to the nearest integer value when rejecting at various Markov times due to the difference in the theoretical optimal policy and the application of the theoretical optimal policy. For example, if the theoretical optimal policy calls for the rejection of 36.8% ($\tau_{\pi }=0.368$) of the population, the application of this theoretical optimal policy may not be feasible as it may be impractical to reject 0.8% of a population unit. Thus, rounding to the nearest percentage integer value may be required $\left(i.e.,\tau_{\pi }=0.37\;\text{where}\;\{\tau \in \mathbb{R}:\tau \in (0,1]\right\}$).

**Table 3:** Probability of discovering a NN model within rank 10 or better (*R*_1–10_).

Neural network model rank^1^	Total population of 100		Total population of 672	
	Markov time as percent of population for rank’s optimal policy^2^	Probability of success discovering best ranks within rank^3^	Markov time as percent of population for rank’s optimal policy^2^	Probability of success discovering best ranks within rank^3^
1	37%	0.3732	37%	0.3703
2	31%	0.5214	29%	0.5172
3	23%	0.6057	27%	0.5983
4	21%	0.6637	23%	0.6507
5	18%	0.7055	20%	0.6922
6	17%	0.7373	19%	0.7250
7	17%	0.7638	16%	0.7476
8	15%	0.7848	16%	0.7695
9	15%	0.8015	16%	0.7863
10	14%	0.8167	15%	0.7990

1 Neural network model ranked by performance; best performing first.

2 Variability in the Markov Time as Percent of Population for Rank’s Optimal Policy between differing total populations is due to rounding to the nearest integer value (whole, non-fractional number). This occurs due to the nature of the Secretary Problem: the optimal policy for a given Rank may not match the reality of decision making. Such as the optimal policy for selecting Rank 1 is to reject 36.8% of the total population; however, it is not possible to reject 0.8% and interview 0.2% of an applicant, thus integer value rounding must occur.

3 Similar to table footnote 2, variability is due to rounding to the nearest integer value when the PDF of the modified version Secretary Problem evaluated at each Markov Time variant for each ordinal Rank in ascending order followed by computing the CDF at each Markov Time variant halting at the optimal policy discovered.

If the researcher does not believe 80% is a high enough probability of success in discovering a NN model within $R_{10}$, Table 4 communicates the requirements to achieve the additional percentage probability of success increases. These effects need to be carefully balanced as the total population *n* grows. While the resulting percentage of the total population to explore (*r/n*) per percentage probability increase decreases overall, the ordinal amount of NN models to build, train, test, and evaluate increases. Thus, if there is an overhead resource cost per a unit of population to “interview” (*r*); the expenditure of these resources must be taken into account as this cost function could become a constraining factor when deciding how much of the search space to explore.

**Table 4:** The NN model rank bound and Markov time as percentage of population required to achieve various probabilities of success.

Probability of success discovering	Total population of 100		Total population of 672	
	Markov time as percent of population for rank’s optimal policy	Neural network model rank bound/as percent of total population	Markov time as percent of population for rank’s optimal policy	Neural network model rank bound/as percent of total population
0.85	11%	13/13%	11%	16/2.3%
0.90	9%	20/20%	7%	28/4.16%
0.95	5%	36/36%	5%	59/8.77%
0.99	2%	72/72%	1%	273/40.62%

### 4.3 CBP application analysis

The second variant of the SP includes the ability to “call back” a previously interviewed candidate at some Markov time determined by the hiring manager. For example, instead of executing the CSP with its optimal policy ($\tau_{\pi }≅0.37$) and then selecting the next best relative candidate before halting: interviewing the minimum number of applicants to establish an informed relative ranking baseline (*n* > 20), ranking the interviewed candidates against the performance threshold and/or objective metric, deciding to continue interviewing or halting all interviews and calling back the highest ranked candidate interviewed for hire. A modern application of the CSP to NAS would likely be executed closely to the modified version of the CSP, GEP, where the researcher has a set a “good enough” performance metric to ensure the balance between the efficient use of limited computational resources, sufficiency in NAS search space exploration, and discovery of a “good enough” neural architecture.

Realistically, a researcher would have the ability to save each evaluated NN model to call upon in the future; thus, modifying the rules of the CSP to an extent where the best “candidate” interviewed can always be “hired” no matter of the Markov time. While typically the limiting computational resource is processing time and power, if storage is the limiting computational resource, then a simple operating procedure of saving the best relative NN model found thus far in memory would suffice.

This “best found” NN model in memory is analogous to the “call back” feature previously described. As better relative NN models are found within the NAS search space, they would replace the NN model occupying the “call back” spot. Logically, as storage is an inexpensive computational resource and the data generated to construct a complete blueprint of a NN model is likely orders of magnitude less than the data used to train each NN model, more than one “call back” position would exist. This allows the researcher to save multiple NN models and explore which NN building block components, like those listed in Table 1, are driving the best performance. This could further help the researcher limit the NAS search space by eliminating certain NN building block components from the NAS design space.

**Figure 8:**
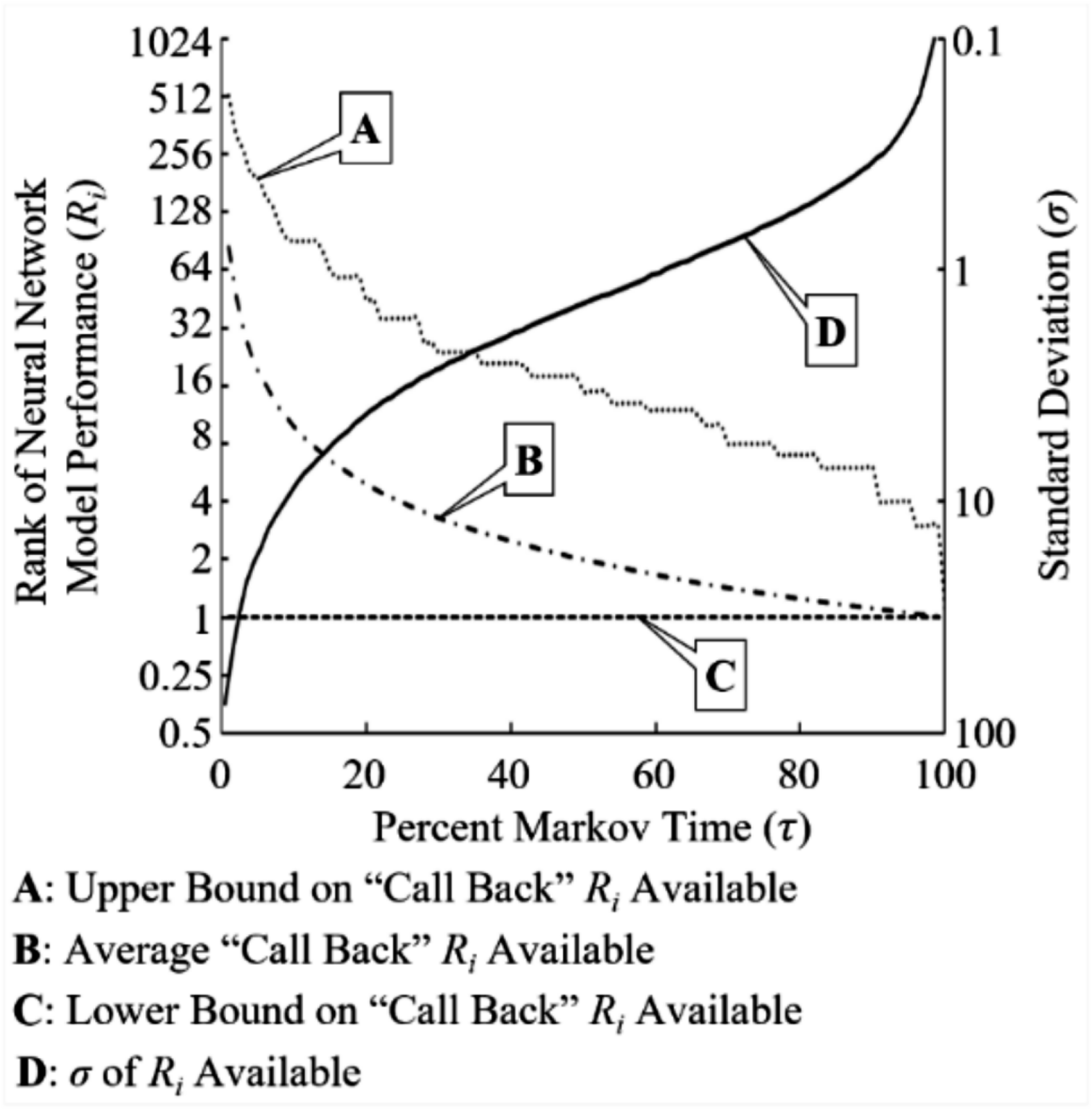
Upper and lower bounds with average NN model rank *i* (*R_i_*) available with the “call back” feature (CBP) implemented along with standard deviation (*σ*) of *R_i_* overlaid.

Figure 8 visualizes these results over the 20,000 cycles of the experiment. Independent of Markov time used, the best NN model rank for possible “call back” selection was $R_{1}$. As expected, the upper bound on the highest (worse) NN model rank for possible “call back” decreases as Markov time increases. The average NN model rank available for “call back” also followed this trend. This average NN model rank available for “call back” is computed as follows:


(7) $${\bar{R}}_{i}^{\mathrm{CBP}}=\tau^{-1}$$

While Equation 7 offers a rudimentary approximation for the average NN model rank available for “call back,” an upper bound approximation is appropriate for more risk adverse researchers. Due to the mechanics of implementing a “call back” feature, if the best-performing NN model (*R*_1_) was not within the rejected population subset (*r/n*) at a given Markov time, the lowest performing NN model rank selected had an upper bound defined as


(8) $$R_{\mathrm{iU}}^{\mathrm{CBP}}={\left(\mathrm{e\tau}\right)}^{-1}\sqrt{n}$$

**Figure 9:**
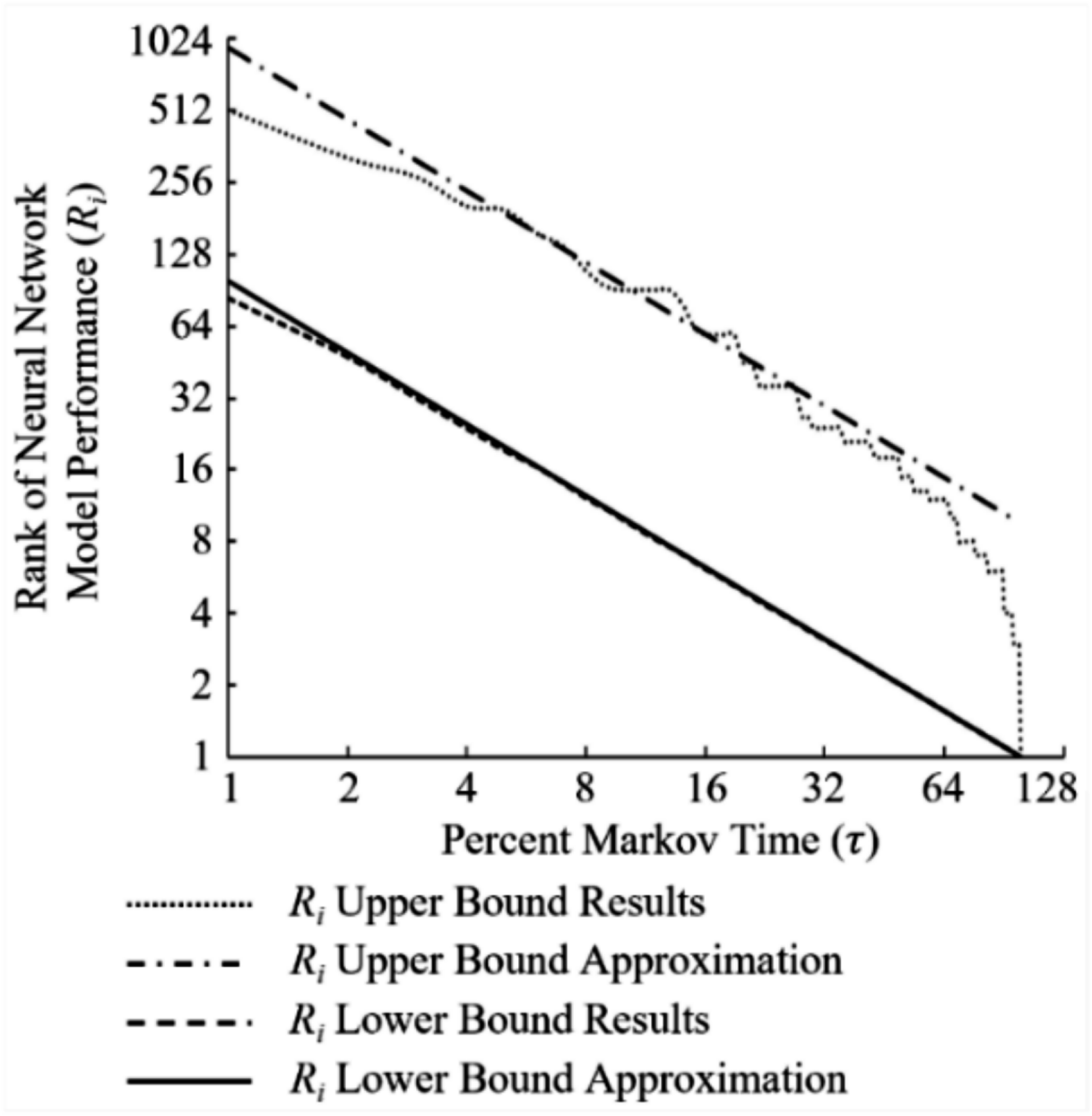
Experimental results vs equation approximation to predict the upper bound and average rank *i* (*R_i_*) selected if rank 1 (*R*_1_) is contained within the non-rejected population subset (BOR) at various Markov times.

Figure 9 shows dependencies represented by Equations 7 and 8 overlaid with experimental data collected. Although these two approximation equations deviate from the experimental data plotted at each end of Figure 9’s *x*-axis, they both provide useful preliminary decision points to researchers who require guarantees, justifications, or confidence metrics to manage communicating progress and performance estimates in NAS search space exploration.

**Figure 10:**
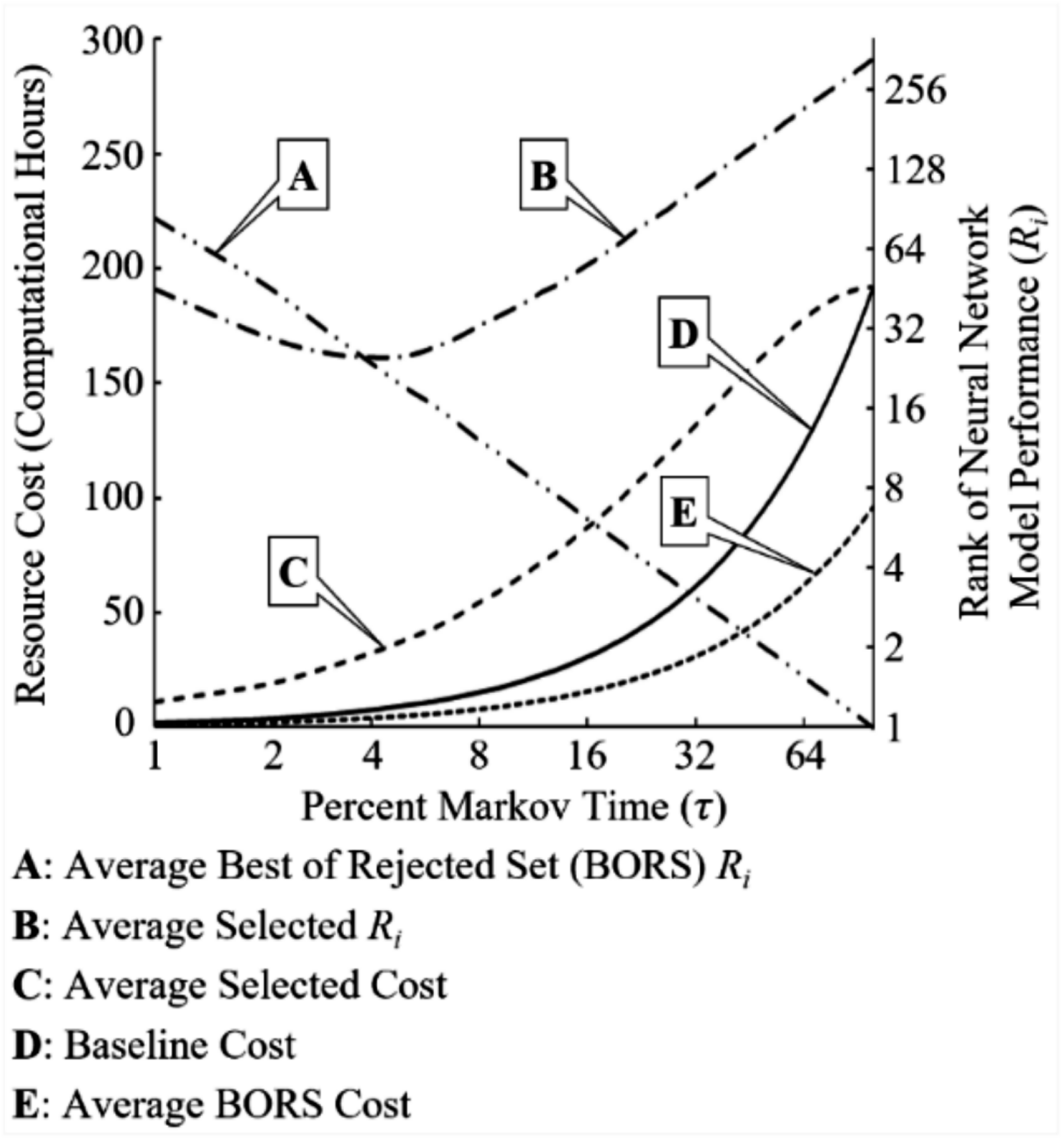
Resource cost requirements per NN model rank *i* (*R_i_*) by Markov time.

The “call back” feature becomes even more powerful when a resource cost is applied to the computational hours required to build, train, test and evaluate each NN model within the NAS search space; these are known as NAS execution activities (NEAs). For the current experiment, the average time to perform these NEAs per NN model per dataset per cycle was 17 min 8 s. Thus, there is an inherent overhead resource cost associated with executing the CSP and its variants. In this particular experiment, a “baseline” overhead cost is associated with each Markov time as percent of total population *n*: seven networks per a single percent Markov time increment results in 1.998 h of computational cost. This resource “penalty” transforms the problem into a balancing act where the goal is to find the highest-performing NN model and halt the NAS function as quickly as possible to minimize computational resource cost. Figure 10 shows how this transformed problem plays out over the entire Markov time spectrum ($\tau_{0.1-1.0}$ where {τ∈ℝ:τ∈(0,1]}). At low Markov times(τ≤0.04) the average BORS rank is higher (worse) than the average selected rank; this quickly reverses asτ>0.04.

Additionally, the required resource cost to execute the CBP vice taking the BORS at each Markov time is more expensive. The “baseline” overhead cost should be seen as a “sunk” cost; that is, a cost required to perform NAS using this method would be levied on the researcher no matter which SP variant is chosen. Furthermore, a researcher should not discount the additional “sunk” cost required to perform NAS setup, integrated development environment configuration, automated software development pipeline orchestration, source code debugging episodes, and results verification and validation activities; these are known as NAS infrastructure support activities (NISAs). NISAs are likely to take more time to complete than executing the NAS effort itself if a single NAS cycle is executed. In this experiment, NISAs took an additional 25 min 42 s per NN model to conclude. This brings the original time per NN model to execute for a single experimental cycle from 17 min 8 s to 42 min 50 s, representing a 149.99% increase in resource cost.

However, NISAs tend to be a fixed cost as compared to the variable cost of NEAs. If multiple NAS cycles are to be executed or the total population is extremely large, the cost of NEAs will undoubtedly be much larger than the cost of NISAs due to cost sharing. As this experiment performed five NAS cycles per dataset, the total experiment NISA cost was 575 h 40 min 12 s. The total experiment NEA cost was 1,918 h 54 min. In terms of cost per NN model per dataset per cycle: NEA cost remains at 17 min 8 s, NISA cost drops to 2 min 34 s. Therefore, it is imperative for researchers to make use of non-manual, automated tools and routines to minimize human-input errors, downtime of NAS phase handoff sequences between tool chains, and flexible NISAs to support reuse for future NAS endeavors.

For the implementation of CBP, it is recommended a researcher endeavor to save the parameters required to rebuild each NN model discovered. If computational resources do not allow for this, saving the highest performing NN model at each state space search step is sufficient. Additionally, it is recommended that the researcher explore a CBP minimum of 4% ($\tau_{\pi }\geq 0.04$) of the NAS search space where *n* > 20. At this Markov time, the average rank of the rejected NN model (BORS) drops below the average rank of the NN model selected (SV) using the CSP rules. Thus, a NN model within the rejected Markov time population will, on average, be a better rank than not utilizing a “call back” feature. This fact, tied with a carefully crafted resource cost function, will aid the researcher in making the best use of computational resources with satisfactory NAS search space exploration coverage.

## 5 Discussion and future work

The application of OST to NAS is a viable solution for researchers to pursue; if and only if the researcher has ensured the NAS methodology fits the constraints and assumptions of the SP rules set up. Key constraints and assumptions include applicants are selected at random to be interviewed, each applicant can be ranked relative to each other as they are interviewed, and the total number of applicants *n* is known and is greater than or equal to 20. Each of these can be overcome with proper NAS setup.

For instance, if the selection and evaluation of each NN model is not done at random, much of the benefit gained from executing the CSP’s optimal policy is negated. This issue is mitigated by either: enumerating each NN model within the search space and then selecting at random from this enumerated list to be built, trained, tested and evaluated, or the code used to build each NN model can select the NN building block components from a determined set at random and then check the resulting combination of the selected NN building block components are of a unique configuration.

Knowing the total number of applicants *n* is an essential element to the CSP’s optimal policy. The simplest way to calculate the total number of neural network models within the NAS search space is to build determined sets of neural network building block components and then apply the Rule of Product in combinatorics. If this NAS design space is determined to be intractable, the researcher may be able to further bound this NAS design space by performing a sensitivity analysis on the neural network building block components. This allows the researcher to remove neural network building block component options that do not significantly contribute to model performance. However, caution must be exercised when conducting this maneuver as it opens up the resulting NAS selection to bias.

The researcher can also choose to apply the solution to the SP and its variants to the time domain aspect of their NAS effort. That is, if the NAS design space is determined to be intractable, the researcher should devote ~37%, ~15%, or ~4% of the NAS effort’s schedule to executing the chosen SP variant’s solution. The researcher should also take care to list all the variables of the NAS endeavor that are dynamic outside of the neural network building block components. These dynamic variables may include items such as artificial neural network structure (class), training parameters (dropout frequency, dataset characteristics (such as resolution size, color channel options, and the like for images), batch size, epochs, etc.), and number of cycles the endeavor will be repeated, if any. All these dynamic variables, if not accounted for and controlled, will have an effect on the results of the NAS endeavor. If the total number of applicants *n* is below 20, a new approach will need to be pursued.

The CSP’s optimal policy is also dependent on the hiring manager’s ability to rank each applicant relative to each other. While this is a simple endeavor for a researcher as each NN model can be assigned a performance score based on its ability to succeed at the desired task (such as image classification), the infrastructure support to automate a “pipeline” for this is not a simple undertaking. To ensure NAS configuration control and efficient use of resources, a seamless process from selecting the neural network model to be built; collecting and cleaning data; building, training, testing, and evaluating NN models; analysis of resulting performance scores; and data recording functions will need to be automated to the fullest possible extent. This will likely involve multiple code bases, programming languages, analysis applications, computational architectures, and data storage formats. These are items of concern for any software intensive project; a researcher will need to understand and identify the limitations, unique behaviors, and special scenarios that may arise not only within the NAS endeavor, but as well as within the tools being used to execute the NAS endeavor. Examples include accounting for latent bugs, deprecated library dependencies, and numerical data limitations between programming languages used; like those of numerical precision, rounding, and cutoff.

Lastly and most importantly, the decisions made during the design phase of the NAS search space are critical. Due to the nature of the SP, there is no guarantee in discovering a high-performing NN model within a NAS search space for the problem it is to be applied to. There is a high degree of confidence in discovering a high-performing NN model within the search space relative to the other NN models contained within the search space. Thus, researchers should pair this paper’s findings with “smart” NAS search space design practices aligned to the problem to be solved.

Table 5 succinctly summarizes the above key constraints and assumptions of applying OST to the SP and its variants for use within NAS endeavors. Table 5 also conveys recommendations and considerations to researchers, by SP variant, for values to use in determining NAS search space coverage in terms of Markov time as a percentage of total population and potential computational resource savings.

**Table 5:** Key constraints, assumptions, recommendations, and considerations for all SP variants.

Key constraints and assumptions for all secretary problem variants	
A	The total number of neural network models within the NAS search space is known
B	The total number of neural network models within the NAS search space is at least 20 (*n* ≥ 20)
C	Neural network models are selected at random to be interrogated and ranked; Every neural network model within the NAS search space has an equal chance at being selected
D	Each neural network model interrogated is relatively ranked from best to worst against only previously interrogated neural network models
E	Every neural network model within the NAS search space can be uniquely ranked; No ties exist

Recommendations and considerations by secretary problem variant			
Secretary problem variant	Recommend optimal policy minimum ($\tau_{\pi }$ or *r/n*)	Computational resource savings (times better)	Considerations
CSP	0.368	2.7x	Interrogating 36.8% of the NAS search space returns the highest probability of success in discovering the best performing neural network model; Interrogating 36.8% of the NAS search space may not be possible for certain total population values of *n*; Rounding up to the nearest population unit integer may be required
GEP	0.15	6.7x	Interrogating 15% of the NAS search space gives an 80% probability of success in discovering a neural network model of Rank 10 or better; As the probability of success in discovering a neural network model of the best Rank with a certain Rank range grows, the required exploration of the NAS search space shrinks resulting in a Rank increase of the best Rank discovered
CBP	0.04	25x	Interrogating at least 4% of the NAS search space returns a better Rank on average with the “call back” feature as compared to the average Rank selected using the CSP rules; Interrogating at least 10% of the NAS search space returns a better Rank on average than the GEP optimal policy of 15%

In the course of this experiment, many interesting artifacts were uncovered and would benefit from further investigation. These fall into three categories: equation refinement, hardware optimization, and integration with state-of-the-art NAS search and evaluation techniques. Equations 4–8 aid researchers in helping to estimate the central questions before executing a SP influenced NAS endeavor. Equation 4 provides researchers with an estimate of the upper bound (rank no worse than) of a NN model’s performance rank if the best performing NN model (*R_1_*) was not rejected when executing the CSP. Equation 5 provides researchers with an estimate of the required amount of search space to explore for a given rank when executing the GEP variant. Similarly, Equation 6 provides researchers with a probability estimate of how likely the discovery a rank of interest is within a rank range. Equations 7 and 8 give researchers the ability to estimate the average rank and the worse rank that could be selected as tied to the amount of search space explored when executing the CBP variant. The importance of these equations to researchers who must justify the expenditure of resources and schedule timelines to conduct NAS efforts and then defend such justifications with data while communicating confidence levels to leadership, cannot be understated.

While Equations 4–8 offer researchers a starting point to help estimate central questions before executing a SP influenced NAS endeavor, it is clear these equations need to be further refined and generalized for different values of *n* to increase their accuracy. Thus far, the equations have only been briefly tested against search space sizes of 100–3,500 stepped at increments of 50 and held to be a guiding heuristic when executing the SP and its variants. In support of this, datasets containing unique, non-repeating random numbers simulating the performance of each NN model for these population sizes were generated along with the results of executing the SP against each of these populations and made publicly available.

Another area for future investigation was uncovered when formulating Equation 4. Dynamic programming was used during the initial stages of the experiment to ensure the results of the CSP could be verified and validated. During this painstaking process of implementing a test-driven software development paradigm in Visual Basic for Applications, the halting of the CSP at various Markov times results in two sets of data: a rejected set of NN models and a not-yet rejected set of NN models. The rejected dataset was searched to reveal the best (highest performing) rank rejected and reveal the best rank remaining within the not-yet rejected dataset. Additionally, the rejected dataset was searched to ascertain if the best NN model of *R_1_* was contained within. Thus, if the NN model of *R_1_* was not within the rejected dataset, the minimum (highest performing) rank within this dataset was subtracted from the minimum (highest performing) rank contained within the not-yet rejected dataset; this revealed how many ranks and positions in memory could possibly be needed to find a better rank. Over the 20,000 experimental cycles, Equation 4 held as an upper bound on potential positions within memory needed for better ranks to occupy. Extending this from the CSP to the GEP and CBP variants may offer valuable design insights when developing computational hardware to implement an optimal stopping algorithm upon which must contend with size, weight, power, and cooling constraints for random search algorithms.

The final area for future investigation is the applying the findings of this paper to other state-of-the-art, random and non-random, NAS search strategies and evaluation techniques to ascertain the possible benefits and limitations of integration. While the solution to the SP and its variants are finite MDP problems and dependent on random selection, it is not clear if a “smartly” designed NAS search space making use of micro search cell-based structures (like that of DARTS; Liu et al., 2019) is paired with a one-shot (Bender et al., 2018), once-for-all (Cai et al., 2019), knowledge distillation (Gou et al., 20121), or other NAS search strategies with this paper’s findings overlaid on top to act as a halting policy would be beneficial. The initial suspicion is that the findings of this paper may only be useful as an optimal policy for random NAS search strategies with robustly designed NAS search spaces. However, empirical evidence is needed.

## 6 Conclusion

The Secretary Problem has potential to help inform researchers when conducting NAS in a manner consistent with the key constraints and assumptions of the famous decision-making riddle. The application of the SP and its variants are both feasible to implement and viable to execute. To do so, however, requires a careful understanding of the SP’s limitations, NAS search space design decisions, and the experimental infrastructure support required to be successful in executing this endeavor.

Results show a researcher would have a high-probability of success in finding the best performing (highest-rank) NN model relative to the performance of other NN models within a NAS search space if they applied the CSP unaltered and explore a minimum of 37% of the NAS search space. However, these results will only materialize if the initial constraints and assumptions of the problems are adhered to.

If the modified or modern version of the SP is executed, significant increases in the probability of successfully finding a relative overall top-ranked NN model will be realized coupled with a drop in required search space exploration to 15 and 4%, respectively. Additionally, the resource cost to explore the NAS search space can be limited resulting in 6.7 and 25 times decrease in computational costs, respectively.

The authors plan on applying the findings of this paper to investigate other state-of-the-art, random and non-random, NAS search strategies, evaluation techniques, and datasets to ascertain the possible benefits and limitations in the future.

## Data Availability

Publicly available datasets were analyzed in this study. This data can be found at: Modified National Institute of Standards and Technology (MNIST) and the Canadian Institute for Advanced Research, 10 Classes (CIFAR-10).
